# Personal Factors Associated with Smoking Among Marginalized and Disadvantaged Youth in Japan: A Strong Relationship Between Smoking and Convenience Store Use

**DOI:** 10.1007/s12529-012-9268-8

**Published:** 2012-09-27

**Authors:** Isao Watanabe, Masako Shigeta, Kaoru Inoue, Daisuke Matsui, Etsuko Ozaki, Nagato Kuriyama, Kotaro Ozasa, Toshiro Yamamoto, Narisato Kanamura, Yoshiyuki Watanabe

**Affiliations:** 1 Epidemiology for Community Health and Medicine, Graduate School of Medical Science, Kyoto Prefectural University of Medicine, 465 Kajii-cho, Kamigyo-ku, Kyoto 602-0841 Japan; 2 Dental Medicine, Graduate School of Medical Science, Kyoto Prefectural University of Medicine, Kyoto, Japan; 3 Epidemiology, Radiation Effects Research Foundation, Hiroshima, Japan

**Keywords:** Smoking, Youth, Disadvantaged, SES, Point of sale, Convenience store

## Abstract

**Background:**

A national survey in Japan reported that the prevalence of smoking among high school students has sharply decreased in recent years. However, the survey only considered students who attended regular high schools (RHSs), and Japan offers part-time high schools (PHSs) that are often attended by academically and socioeconomically disadvantaged youth.

**Purpose:**

Therefore, we examined the smoking prevalence and smoking-related factors among PHS students.

**Method:**

A self-administered questionnaire-based survey was conducted at six PHSs. The subjects included 540 enrolled students aged 15 to 18 years. The questionnaire included items on smoking status, smokers in the family, frequency of convenience store use, lifestyle behaviors, and health awareness. Logistic regression analysis was used to identify factors that were significantly associated with smoking.

**Results:**

A total of 45.6 % of students had smoking experience, and 29.3 % were smokers. For males and females, the smoking prevalence was about 3 and 7–12 times higher, respectively, than that reported in the national survey. The factors found to be significantly associated with smoking included having a smoker in the family, experience with drinking alcohol, and using convenience store daily (odds ratio [OR] = 12.5) or sometimes (OR = 3.63). There was a significant dose–response relationship between smoking and convenience store use.

**Conclusion:**

The smoking prevalence among PHS students was remarkably higher than that among RHS students. These findings suggest that marginalized and disadvantaged youth should be targeted for tobacco control, and intervention is needed to protect youth from tobacco sales and advertising at convenience stores.

## Introduction

Youth smoking is an extremely serious public health issue. Tobacco use and nicotine addiction generally begins during youth and continues through adulthood, and smokers who become addicted in their youth have the highest risk of tobacco-related disease [1–6]. In particular, smoking among marginalized and disadvantaged youth is considered to be an important health concern, as smoking starts earlier among low socioeconomic status (SES) groups [3–5], and earlier onset is associated with heavier tobacco use and addiction [2–4].

The Global Youth Tobacco Survey (GYTS) indicated that there are major differences in youth smoking across different countries [7]. According to the Tobacco Atlas (third edition) [2], which used the GYTS as its major source, smoking is seen among more than 30 % of boys in Papua New Guinea, Timor-Leste, and Germany, and more than 30 % of girls in Spain, Germany, and Austria. The prevalence of smoking among Japanese youth was not reported in either the Tobacco Atlas or the GYTS, but an independent survey of smoking among Japanese high school students was begun in 1996 and continues into the present. The results of this survey indicated that the prevalence of smoking among high school students (10th–12th grades) in Japan in 2008 was 8.6 % for males and 4.6 % for females, which was low by global standards [8, 9]. Furthermore, the prevalence of smoking (i.e., having smoked at least one cigarette in the 30 days preceding the survey) among high school students sharply decreased by about two thirds from 1996 to 2008. For males, the prevalence was 30.7 % in 1996, 29.9 % in 2000, 15.9 % in 2004, and 8.6 % in 2008; for females, the prevalence was 12.6 % in 1996, 19.1 % in 2000, 8.2 % in 2004, and 4.6 % in 2008 [8, 9]. This survey was sufficiently large and was conducted systematically by a government-associated research group. However, it considered only students who attended regular high schools (RHSs).

In Japan, the first 9 years of education are compulsory, and 3–4 % of ninth graders do not progress to high school (tenth grade), and 8–9 % of enrollees drop out during high school [10]. In addition, not all enrollees attend RHSs; some attend part-time high schools (PHSs), which began with the enactment of the School Education Act in 1947 and were established for “young workers who wish to pursue their upper secondary studies in a flexible manner in accordance with their own needs.” [11]. Most PHSs are open weekday evenings and offer 4-year curricula, in contrast to the 3-year curricula at RHSs. PHS students constituted 2.6 % of all high school students in 2000 and 3.5 % in 2010 [10], reflecting an increase that could be due to the poverty issues arising from the long-term recession in Japan.

The PHSs of Japan operate under a unique framework, but there are three types of alternative high schools (AHSs) in the USA: Type I schools provide innovative programs and nontraditional administrative organizations; Type II schools are “last-chance programs” to which students are assigned, usually as an alternative to expulsion or jail; and Type III schools are for remediation or rehabilitation in academic or social/emotional areas, or both, with the goal of enabling students to return to regular programs [12]. PHSs in Japan are somewhat similar to Type III AHSs in the USA. The International Union for Health Promotion and Education (IUHPE) has emphasized the health of out-of-school youth, as they are often vulnerable to disease and high-risk behaviors, and are notoriously hard to reach through conventional educational media [13]. Both the PHS system in Japan and the Type III AHS system in the USA seem to have the advantage of keeping youth in school, potentially decreasing the proportion of out-of-school youth in the respective societies.

Previous studies have suggested that students outside of the mainstream educational system have a higher smoking prevalence than students in ordinary high schools. Reports from the USA, Hong Kong and New Zealand showed that the prevalence of smoking was high in AHSs [12, 14, 15]. Recently, PHSs serve youth who are unable to attend RHSs for academic or economic reasons. In Japan, PHS students often have a disadvantage in terms of school performance, and studies have indicated that lower school performance is related to a higher level of smoking experience [3, 16, 17]. Unlike the situation in RHSs, however, the shortage of school time in PHSs means that the needed health education against risky behaviors is not included in the basic curricula. In 2007, Shimane and Wada [18] conducted a survey of three PHSs and found that smoking and alcohol consumption were very common. Therefore, the smoking status among PHS students in Japan requires urgent investigation.

We also question how the tobacco sales environment might affect at-risk youth. National-level tobacco control policies are extremely weak in Japan, largely because the Japanese government has invested in a tobacco company (Japan Tobacco Inc.) and arranges for retired bureaucrats to work in the company [19, 20]. Additionally, Japan has been subjected to the shrewd marketing tactics of overseas tobacco industries since market liberalization in 1985 [19, 21]. Although the prevalence of smoking in Japan has decreased overall, measures to reduce smoking in low SES groups have been insufficient.

In an effort to prevent youth smoking, Japan enacted a law called the Act on Prohibition of smoking by Minors (<20 years old) in 1900. However, this law did not produce the intended effect [8] because most minors bought tobacco through vending machines during the 1990s. To discourage this problem, the system of adult-recognition IC cards (taspo) for vending machines was introduced in 2008. Cigarettes now only are purchased upon presentation of the card at vending machines. A photo and a copy of their official identification such as their driving license are required to apply for the card. However, this system had only a very limited effect [22, 23], as the route of tobacco sales to minors shifted from vending machines to convenience stores.

In 2011, Japan Tobacco Inc. reported that 60 % of their tobacco products were sold at convenience stores, and announced that they would promote investment in “point of sale (POS)” [24]. Japan has more than 44,000 convenience stores, approximately 94 % of which are open 24 h per day, 365 days per year (Japan Franchise Association Data 2008), and most of which sell tobacco and alcohol. Moreover, overseas tobacco industries have invaded Japan in recent years, expanding their share of Japanese tobacco sales from 2.4 % in 1985, 15.9 % in 1990, 21.2 % in 1995, 26.7 % in 2002, 35.2 % in 2005 and 35.9 % in 2010 [21, 25]. These industries have taken advantage of their experience in marketing to younger people, and have inundated Japan with smart packaging, direct mailings, cigarette offers, gifts with purchases, promotional items, and so on. Today, convenience stores in Japan are filled with POS tobacco displays. Moreover, these industries have penetrated beyond the Japanese market to those of other Asian countries [26]. Therefore, the influence of these marketing activities on smoking behavior among young people should be investigated, in the hopes of shedding light on this potentially international problem.

Here, our first objective was to compare the prevalence of smoking among students of six PHSs versus the RHS data from the national survey, in order to examine the smoking status of marginalized and disadvantaged youth in Japan. Our second objective was to analyze factors related to smoking among PHS students, focusing on convenience store use in consideration of the recent shift in tobacco sales. Our findings may prove useful in the future development of measures to reduce the prevalence of smoking among vulnerable students.

## Methods

### Subjects and Study Period

The anonymous self-administered questionnaire survey was administered from April 2008 to March 2009 at six PHSs (1,060 registered students) in the Kansai area that had requested smoking prevention classes. The survey was administered to the 697 students (65.8 % coverage rate) who were present on the day of the survey and was distributed with the cooperation of homeroom teachers. The purpose was explained, and students were assured that school personnel would not see the individual responses. A response was considered to constitute consent to participate in this study. After the responses were collected, they were immediately put into sealed envelopes and returned to the researchers.

Among the 697 respondents, 19 were eliminated due to lack of information regarding sex, age, smoking experience, or smoking status (valid response rate, 97.3 %), and 138 respondents who were 19 years or older were eliminated to make the results comparable to the data in the national survey. A total of 540 responses were therefore used for our analysis. Subjects were classified by age, because grade levels do not correspond exactly with age in PHSs. For comparisons with RHS students, we considered age 15 at a PHS to correspond to tenth grade at an RHS, age 16 at a PHS to correspond to 11th grade at an RHS, and ages 17 and 18 at a PHS to correspond to 12th grade at an RHS.

### Content of Study

#### Anonymous Self-Administered Questionnaire

The survey collected information on sex, age, smoking experience, smoking status, and smokers in the family. Smoking every day was classified as “smoking daily.” Smoking one or more cigarettes in the 30 days preceding the survey but not smoking every day was classified as “smoking occasionally.” Students who had smoked daily or occasionally during the 30 days preceding the survey were defined as “smokers.” Students who had not smoked during the 30 days preceding the survey, with or without prior smoking experience, were defined as “non-smokers.”

The lifestyle-related items examined herein were hours of sleep per night, bedtime and waking time, alcohol drinking experience, breakfast and snack habits, daily time spent watching television, studying at home, Internet use, and convenience store use. Additionally, we asked about factors associated with health awareness. Consumption of breakfast and snacks were classified as “Every day,” “Sometimes,” and “Don’t eat (No.)” Convenience store use were similarly classified as “Every day,” “Sometimes,” or “Don’t go (None.)” The three categories for television watching were “≧3 hours,” “0–3 hours,” and “Don’t watch (0 hour.)” The two options for studying at home and Internet use were “Study/Use (Yes,)” and “Do not study/Do not use (No.)” respectively. The items concerning health awareness were mainly taken from the Health Locus of Control Questions [27], and also included some other items, including, “Luck plays a big part in determining if you will get sick,” “No matter what I do, if I am going to get sick, I will get sick,” “If I take care of myself, I can avoid illness,” “I am healthier than others,” “I am concerned about my physical strength,” and “I often think about my health.” Four responses were available for each health awareness item: “Agree strongly,” “Agree somewhat,” “Disagree somewhat,” and “Disagree strongly.”

### Analytic Methods

The *χ* ^2^ test was used to compare the prevalence of smokers between males and females, and the Student’s *t*-test was used to compare hours of sleep per night between smokers and non-smokers. Odds ratios (ORs), 95 % confidence intervals (CIs), and p values were calculated using a logistic regression analysis, in which smoking (daily or occasionally) was the dependent variable and lifestyle behaviors, smokers in the family, and health awareness were taken as independent variables. Trend tests for dose-response were used to evaluate the effects of the three-category lifestyle behaviors. A *p* value less than 0.05 was considered significant. SPSS 18.0J for Windows (SPSS; Japan Inc.) was used for all statistical analyses.

### Ethical Considerations

The ethics board of the Kyoto Prefectural University of Medicine approved the study protocol.

## Results

### Characteristics of Subjects

Table 1 shows the demographic characteristics of the enrolled PHS students. The mean age was 16.3 ± 1.02 years; there were 138 fifteen-year-olds, 204 sixteen-year-olds, 111 seventeen-year-olds, and 87 eighteen-year-olds. There were 314 males (58.1 %) and 226 females (41.9 %).

**Table 1:** Characteristics of subjects

	Males	Females	Total
Total number	314	226	540
Mean age ± SD	16.3 ± 1.04	16.2 ± 0.98	16.3 ± 1.02
Age distribution (years)			
15	77 (24.5 %)	61 (27.0 %)	138 (25.6 %)
16	117 (37.3 %)	87 (38.5 %)	204 (37.8 %)
17	61 (19.4 %)	50 (22.1 %)	111 (21.1 %)
18	59 (18.8 %)	28 (12.4 %)	87 (16.1 %)
Smoking experience			
Yes	140 (44.6 %)	106 (46.9 %)	246 (45.6 %)
No	171 (54.5 %)	116 (51.3 %)	287 (53.0 %)
No answer	3 (1.0 %)	4 (1.8 %)	7 (1.3 %)
Smoking status			
Smoking daily^a^	83 (26.4 %)	62 (27.4 %)	145 (26.9 %)
Smoking occasionally^b^	3 (1.0 %)	10 (4.4 %)	13 (2.4 %)
Ex-smoking^c^	53 (16.9 %)	31 (13.7 %)	84 (15.6 %)
Never smoked	171 (54.5 %)	116 (51.3 %)	287 (53.0 %)
No answer	4 (1.2 %)	7 (3.1 %)	11 (2.0 %)

^a^Smoking daily: smoking every day of the 30 days preceding the survey

^b^Smoking occasionally: smoking at least one cigarette in the 30 days preceding the survey

^c^Ex-smoking: had not smoked during the 30 days preceding the survey, but had prior smoking experience

### Smoking Status

Table 1 shows the proportion of smoking experience (smoking even once in an individual’s lifetime) and smoking prevalence (smoking daily or occasionally in the 30 days preceding the survey) for males and females. The proportion of smoking experience was 44.6 % for males and 46.9 % for females, which was not significantly different. The smoking prevalence was 27.4 % for males and 31.8 % for females, which also was not significantly different.

**Fig. 1:**
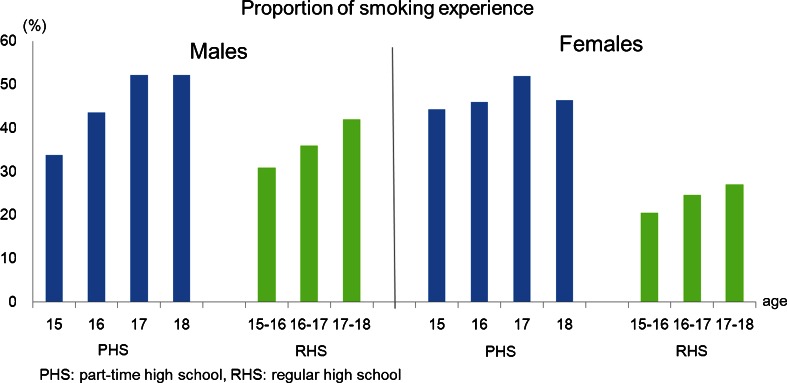
Comparison of the proportion of smoking experience between the part-time high school students and regular high school students (data on RHS students was from a nationwide cross-sectional survey in 2004)

**Fig. 2:**
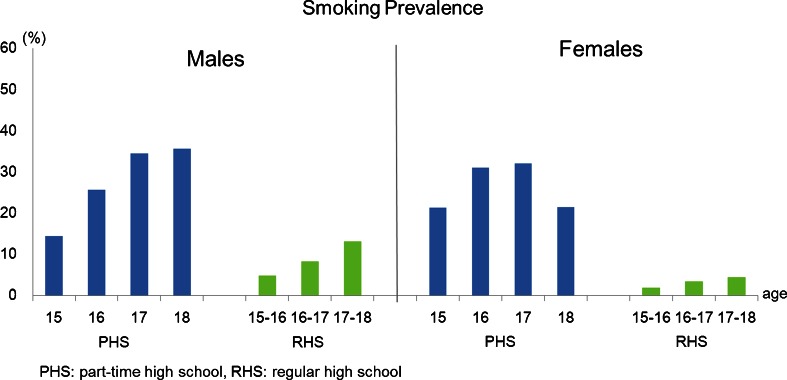
Comparison of the smoking prevalence (daily) between the part-time high school students and regular high school students (data on RHS students was from a nationwide cross-sectional survey in 2004)

Figure 1 shows that the overall proportions of smoking experience among PHS students were much higher than those previously found in RHS students (36.0 % for males and 24.0 % for females) [8]. Figure 2 indicates that the prevalence of daily smoking among 15-year-old male PHS students was three times higher than that of comparable RHS students, whereas the prevalence of daily smoking among 15-year-old female PHS students was 12.5 times higher than that of comparable RHS students.

### Smoking Status and Sleep Behavior

Table 2 shows the relationship between smoking status and sleep behavior. Overall, smokers had significantly fewer sleeping hours than non-smokers (6:55 ± 2:05 vs. 7:21 ± 1:48 hours, *p* = 0.03). The mean bedtime was 2:09 am ± 2:07 hours for smokers and 1:48 am ± 2:19 hours for non-smokers, and the mean time of waking was 9:05 am ± 2:48 hours for smokers and 9:05 am ± 2:34 hours for non-smokers. The differences in these bedtimes and waking times were not significant.

**Table 2:** Sleeping status of smokers and non-smokers

	Males (*n* = 302)		Females (*n* = 213)		Total (*n* = 515)	
	Smokers^a^ (*n* = 83)	Non-smokers^b^ (*n* = 219)	Smokers (*n* = 69)	Non-smokers (*n* = 144)	Smokers (*n* = 152)	Non-smokers (*n* = 363)
Mean total sleep ± SD (h)	6:58 ± 2:07	7:21 ± 1:47	6:52 ± 202	7:24 ± 1:54	6:55 ± 2:05	7:21 ± 1:48
	*p* = 0.17		*p* = 0.09		*p* = 0.03	
Mean bedtime ± SD	2:10 am ± 2:14	1:49 am ± 2:35	2:08 am ± 1:59	1:43 am ± 1:49	2:09 am ± 2:07	1:48 am ± 2:19
	*p* = 0.26		*p* = 0.16		*p* = 0.09	
Mean waking time ± SD	9:09 am ± 2:56	9.03 am ± 2:28	9:02 am ± 2:26	9:07 am ± 2:27	9:05 am ± 2:48	9:05 am ± 2:34
	*p* = 0.97		*p* = 0.80		*p* = 0.89	

^a^Smokers: students who had smoked daily or occasionally during the 30 days preceding the survey

^b^Non-smokers: students who had not smoked during the 30 days preceding the survey, with or without prior smoking experience

### Smoking Status, Smokers in the Family, and Lifestyle Behaviors

Table 3 shows the relationship of smoking status with smokers in the family and lifestyle behaviors. Smoking was significantly more common in students from families with smokers (OR = 2.54; 95 % CI, 1.47–4.37) and there was an extremely strong relationship between smoking and experience with drinking alcohol (OR = 42.1; 95 % CI, 10.3–173). A dose–response analysis indicated that there was a strong positive association between smoking and convenience store use (every day, OR = 12.5; 95 % CI, 4.34–36.3; sometimes, OR = 3.63; 95 % CI, 1.26–10.4; *p* for trend <0.01). The smoking prevalence was significantly greater in males who did not eat breakfast (OR = 2.05; 95 % CI, 1.02–4.10), and lower in males who studied at home (OR = 0.56; 95 % CI, 0.31–0.94). Among females, smoking was inversely related with the amount of time spent watching television (≧3 h, OR = 0.23, 95 % CI 0.09–0.61; 0–3 h, OR = 0.33, 95 % CI 0.12–0.87) and those who used the Internet (OR = 0.51; 95 % CI, 0.28–0.91).

**Table 3:** Association of lifestyle habits and smoking status

		Males		Females		Total		*p* for trend^b^
		*n*	OR (95 % CI)^a^	*n*	OR (95 % CI)	*n*	OR (95 % CI)	
Smokers in the family	Yes	240	2.40 (1.19–4.83)*	179	2.69 (1.13–6.42)*	419	2.54 (1.47–4.37)**	―
	No	69	1.00	40	1.00	109	1.00	
Skipping breakfast	Everyday	124	2.05 (1.02–4.10)*	83	1.13 (0.56–2.28)	207	1.51 (0.93–2.45)	0.061
	Sometimes	117	1.27 (0.62–2.62)	74	0.79 (0.38–1.66)	191	0.98 (0.59–1.63)	
	No	68	1.00	60	1.00	128	1.00	
Eating snacks	Everyday	85	1.78 (0.83–3.81)	53	0.70 (0.27–1.84)	138	1.29 (0.72–2.33)	0.35
	Sometimes	163	1.38 (0.68–2.79)	138	0.62 (0.26–1.46)	301	1.07 (0.63–1.82)	
	No	60	1.00	26	1.00	86	1.00	
Drinking experience	Yes	209	58.1 (7.94–425)**	175	25.3 (3.39–188)**	384	42.1 (10.3–173)**	―
	No	91	1.00	38	1.00	129	1.00	
TV watching time/day	≧3 h	127	0.72 (0.35–1.50)	100	0.23 (0.09–0.61)**	227	0.52 (0.29–0.90)**	0.005
	0–3 h	123	1.04 (0.51–2.13)	92	0.33 (0.12–0.87)*	215	0.74 (0.43–1.29)	
	0 h	50	1.00	21	1.00	71	1.00	
Studying at home	Yes	105	0.56 (0.31–0.94)*	56	0.93 (0.48–1.79)	161	0.66 (0.43–1.01)	―
	No	195	1.00	151	1.00	346	1.00	
Using the Internet	Yes	151	1.21 (0.73–2.00)	110	0.51 (0.28–0.91)*	261	0.84 (0.57–1.22)	―
	No	150	1.00	99	1.00	249	1.00	
Convenience store use	Every day	91	10.9 (3.12–37.7)**	81	13.8 (1.70–111)**	172	12.5 (4.34–36.3)**	0.001
	Sometimes	170	3.77 (1.10–12.9)**	119	3.23 (0.40–26.1)	289	3.63 (1.26–10.4)**	
	None	41	1.00	12	1.00	53	1.00	

^a^OR: odds ratio of smoking (daily or occasionally) relative to non-smoking (ex-smoking or never smoked) and 95 % confidence interval

^b^For three categories with ordinal scale

*p < 0.05

**p < 0.01

### Smoking Status and Health Awareness

Table 4 shows the relationship between smoking status and health awareness. Males were more likely to smoke if they agreed with the statement, “Luck plays a big part in determining if you will get sick” (OR = 2.11; 95 % CI, 1.27–3.52). None of the other associations between smoking status and health awareness were significant.

**Table 4:** Association of health awareness and smoking status

	Males			Females			Total		
	Yes	No	OR (95 % CI)^a^	Yes	No	OR (95 % CI)	Yes	No	OR (95 % CI)
Luck plays a big part in determining if you will get sick.	153	156	2.11 (1.27–3.52)*	101	118	0.83 (0.47–1.47)	254	274	1.39 (0.95–2.01)
No matter what I do, if I am going to get sick, I will get sick.	192	117	1.59 (0.93–2.71)	141	78	1.06 (0.59–1.91)	333	195	1.34 (0.90–1.98)
If I take care of myself, I can avoid illness.	229	80	0.68 (0.39–1.18)	168	48	1.25 (0.62–2.51)	397	128	0.88 (0.57–1.35)
I am healthier than others.	143	165	1.07 (0.65–1.77)	97	117	0.62 (0.35–1.10)	240	282	0.84 (0.58–1.23)
I am concerned about my physical strength.	131	177	1.34 (0.81–2.21)	59	156	1.06 (0.56–1.99)	190	333	1.17 (0.79–1.72)
I often think about my health.	136	173	0.73 (0.44–1.21)	65	151	0.94 (0.50–1.74)	201	324	0.78 (0.53–1.15)

^a^OR: odds ratio of smoking (daily or occasionally) relative to non-smoking (ex-smoking or never smoked) and 95 % confidence interval

*p < 0.05

## Discussion

The CDC has recommended that community-based interventions aimed at the reduction of smoking should focus on four goals: (1) preventing initiation among youth and young adults, (2) promoting quitting among adults and youth, (3) eliminating exposure to second smoke, and (4) identifying and eliminating tobacco-related disparities among population groups [5]. All of these items are relevant to smoking among marginalized and disadvantaged youth, since numerous lines of evidence indicate that smoking starts earlier in the lower socioeconomic groups [3–5, 28–30] and earlier onset is related to more difficulty in quitting [4–6, 31]. Young smokers also risk continuous exposure to environmental tobacco smoke, because parental smoking is strongly associated with smoking among children [2–5].

The proportions of smoking experience among PHS students obtained herein were much higher than those previously found in RHS students. However, the proportions were slightly lower than those previously reported in PHS students by Shimane and Wada [18] (60.6 % for males and 51.5 % for females in 2006), but appear reasonable considering the recent overall decrease in smoking prevalence in Japan. A 1997 survey found that the proportion of smoking experience was 92 % (84.8 % for males, 97.3 % for females) in American AHSs, while that among RHS students was only 70.2 % for both sexes combined [12]. Similarly, reports from New Zealand and Hong Kong showed that students outside of the mainstream educational system smoked more than students in ordinary high schools [14, 15].

The prevalence of daily smoking among female PHS students was remarkably higher than that of comparable RHS students. A survey in the USA found that the prevalence of current smoking at AHSs was 1.9 times higher than that at RHSs [12], while a study of AHSs in New Zealand reported a higher smoking prevalence in females (69.9 %) than males (52.8 %) [15]. Taken together, these results suggest that a non-mainstream education generally has a greater effect on smoking by females than by males. Based on the results of its national survey, the Japanese government believes that current measures related to youth smoking are sufficient. However, we are convinced that these large disparities between RHS and PHS students will have serious health consequences in the future.

The proportion of our PHS students who had smokers in the family was extremely high, at 79.6 %. The national survey of RHSs reported that approximately 45 % of students had fathers who smoked, and 15 % had mothers who smoked [8]. In our study, we did not specifically ask which family members smoked. However, from the 1980s to the mid-1990s, the overseas tobacco industries targeted working-class women [19, 32], and many young Japanese women started to smoke. Research on a nationally representative adult sample of Japanese in 2001 indicated that there was a relationship between smoking and low SES, and the risk for smoking was high in working adult women in Japan [33, 34]. Thus, it is possible that smoking by mothers influences the behavior of their children. We suggest that smoking in Japan could be recognized as an issue with a “generational chain,” as is currently done in Western countries.

In the first half of the 20th century, many Japanese were very poor on average, and could not easily purchase cigarettes. In the second half of the century, most Japanese people enjoyed remarkable economic growth, and 82.3 % of males and 15.7 % of females smoked in 1975 (Japan Tobacco Inc. survey; http://www.health-net.or.jp/tobacco/product/pd090000.html). In other words, most males smoked and most females did not, regardless of SES. During the three decades after World War II, there was a notable reduction in the difference in income between the richest and poorest people. There were also remarkable improvements in life expectancy in Japan, in spite of the high prevalence of smoking [35]. At the turn of the present century, the smoking prevalence among Japanese males began to gradually decrease, reaching 36 % in 2011. During this same period, the smoking prevalence among Japanese females decreased less dramatically, to 12.0 %. Nevertheless, due to the high smoking prevalence among males during the late 20th century, the population-attributable fraction of all-cause mortality due to smoking today is huge at 27.8 % for males and 6.7 % for females [36].

At the same time that the smoking prevalence decreased in Japan, Japanese society was transforming from an equitable society into a competitive society, due to the collapse of the economic bubble in the early 1990s. Japan was recently ranked fourth among the 30 Organization for Economic Co-operation and Development (OECD) countries in terms of relative poverty rate (meaning people who live on less than half median incomes) [37]. These background details and the results from our survey suggest that marginalized and disadvantaged youth have most recently been smoking, which could lead to the serious expansion of health disparities in the near future. Some developing countries, especially Asian countries, currently face both the economic growth and pandemic smoking [1, 2] previously seen in Japan. Thus, investigations into the association between transformation and smoking status should be regarded as critical not just in Japan, but worldwide.

Our survey included questions on health awareness, in order to identify factors related to smoking. The only significant association was for smoking in males who agreed that “Luck plays a big part in determining if you will get sick.” Consistent with this finding, Eiser et al. [27] conducted a survey of 10,579 eleven- to sixteen-year-old students and concluded that, “Smokers, compared with non-smokers, showed less belief in the importance of 'powerful others' or 'personal control' but more belief in the importance of 'chance' as an influence on health outcomes.” Nonetheless, our results suggest that health awareness might be less important than lifestyle and environmental factors with regard to smoking.

We also found that smoking among PHS students was associated with a short sleep time, skipping breakfast, experience with drinking alcohol, and not studying at home. Other reports found a higher prevalence of alcohol drinking in students who were not receiving mainstream educations [12, 15, 18], suggesting a need for future investigations into the alcohol consumption of PHS students. Previous studies have also identified relationships between smoking and sleep/breakfast in adolescents [38–40]. Although the results varied, it is to be expected that smoking would influence or confound various lifestyle behaviors because of the addictive nature of tobacco.

We further found that the prevalence of smoking was lower among females and in all students who watched at least 3 h of television at home, lower in males who studied at home, and lower in females who were internet users. These results may be explained by the greater smoking tendency among people who socialize with friends outside the home [2–4, 29]. Osaki et al. [8] concluded that an increase in the number of students without friends might have contributed to the decrease of smoking in Japan. In light of our findings, we speculate that while RHS students may have gradually developed more independence due to material wealth, a more traditional collectivism may persist among PHS students.

Notably, we found a significant dose–response relationship between smoking and convenience store use. Henriksen et al. reported that smoking prevalence was higher in people who visited convenience stores, liquor stores, and small grocery stores at least once per week [41]. The PHSs examined in the present study were all evening schools, and many of their students used convenience stores as places to eat or meet other people on the way home from school. Thus, friendship networks might influence smoking behavior, as reported by Alexander et al. [42].

In addition to these friendship networks, convenience stores are loaded with tobacco advertisements, including gaudy POS displays in front of and behind the counters. It has been reported that tobacco advertising plays a strong role in persuading young people to start smoking [16, 17, 43–46]. In Japan, most convenience stores sell cigarettes, and tobacco advertising is not regulated. Accordingly, we believe that the influence of convenience store POS displays on smoking behavior among young people should be examined.

The present study has several limitations. First, this was a cross-sectional study, and thus could not identify causal relationships. It is therefore possible that smokers frequently use convenience stores to buy cigarettes, or that their frequent convenience store use induce them to buy cigarettes. A second limitation is that our survey did not include questions related to SES (household income, parental education, parental occupation), motivation for the student to start smoking, and the age at which the student started smoking. Although these are important factors in the assessment of smoking status, we preferred a short and simple questionnaire because PHS students are often academically disadvantaged and could have difficulty answering more detailed questions. A third limitation is that the number of subjects was relatively small, and we were unable to simultaneously survey control subjects in the same region. Finally, this was a school-based study, in which questionnaires were given to students who attended school. Smokers may have a poorer attendance rate, so school-based studies may underestimate smoking prevalence. In the PHSs, approximately 26 % are absent daily, and more than 16 % of PHS students drop out per year. Thus, even though our response rate was high, we probably underestimated the prevalence of smoking in PHS students.

## Conclusion

The prevalence of smoking was extremely high among Japanese PHS students. Smoking was associated with lifestyle behaviors, such as skipping breakfast, short sleep duration, and alcohol drinking, as well as with the presence of a smoker in the family. There was a strong relationship between smoking and convenience store use. In Japan, the health disparity between the rich and poor is increasing, and smoking is becoming a major health issue for socially or economically marginalized youth. Thus, marginalized and disadvantaged youth should be targeted more heavily for tobacco control, and we suggest that intervention is needed to protect youth from tobacco sales and advertisements at convenience stores.
